# Exploiting breath figure reversibility for *in situ* pattern modulation and hierarchical design†

**DOI:** 10.1039/d2sm01650h

**Published:** 2023-03-14

**Authors:** Francis J. Dent, David Harbottle, Nicholas J. Warren, Sepideh Khodaparast

**Affiliations:** a School of Mechanical Engineering, University of Leeds Leeds LS2 9JT UK s.khodaparast@leeds.ac.uk; b School of Chemical and Process Engineering, University of Leeds Leeds LS2 9JT UK

## Abstract

The breath figure (BF) method employs condensation droplets as dynamic templates for patterning polymer films. In the classical approach, dropwise condensation and film solidification are simultaneously induced through solvent evaporation, leading to empirically derived patterns with limited predictability of the final design. Here we use the temporally arrested BF methodology, controlling condensation and polymerisation independently to create diverse BF patterns with varied pore size, arrangement and distribution. External temperature control enables us to further investigate and exploit the inherent reversibility of the phase change process that governs the pattern formation. We modulate the level of subcooling and superheating to achieve subsequent regimes of condensation and evaporation, permitting *in situ* regulation of the droplet growth and shrinkage kinetics. The full reversibility of the phase change processes joined with active photopolymerisation in the current approach thus allows arresting of predictable BF kinetics at intermediate stages, thereby accessing patterns with varied pore size and spacing for unchanged material properties and environmental conditions. This simultaneous active control over both the kinetics of phase change and polymer solidification offers affordable routes for the fabrication of diverse predictable porous surfaces; manufacture of monolithic hierarchical BF patterns are ultimately facilitated through the advanced control of the BF assembly using the method presented here.

## Abstract 2

The breath figure method employs self-assembled condensation droplets as dynamic templates for patterning polymer films. By exploiting the reversibility of the phase change processes, we achieve *in situ* control towards generating diverse patterns.

## Graphical abstract

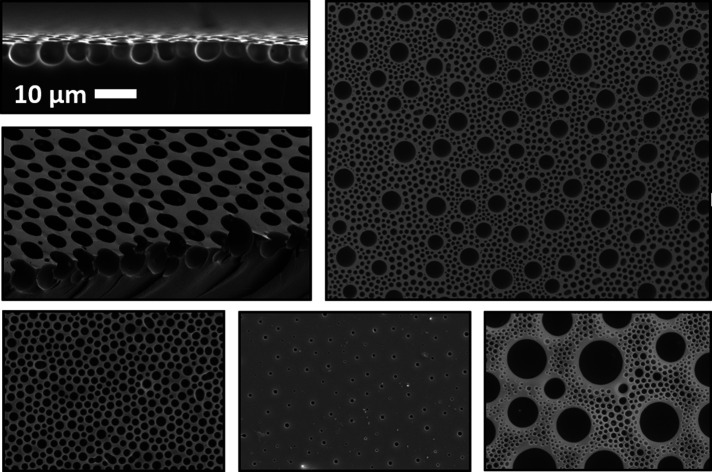

## 1 Introduction

The costly and non-scalable nature of conventional, top-down fabrication methods are fundamental barriers to the ubiquitous application of functional micro/nanopatterned surfaces.^1,2^ In nature, complex surface structures across several length scales often emerge *via* inherently energy-efficient and instability-driven self-assembling mechanisms, offering inspiration for the realisation of intricate designs.^3–5^ Bio-inspired patterning approaches harnessing molecular and interfacial interactions thus provide solutions to high-throughput and low-cost manufacturing of small features over larger surface areas.^6–8^ Full exploitation of such biomimetic fabrication methods towards predictive surface patterning, however, relies upon the understanding and taming of the underlying complex multiphysics phenomena.^9^ Here, we explore the possibility of tuning the surface architecture achieved through the well-known breath figure (BF) templating approach by exploiting the reversibility of the phase change process that governs the operation.

The BF method harnesses the growth and self-assembly of condensation droplets to induce honeycomb-patterns (HCP) on the surface of polymer films.^10^ This maskless, bottom-up surface patterning approach, herein described as the classical BF approach, uses long chain and end-functionalised polymers dissolved in a highly volatile solvent under the presence of a humid environment.^11,12^ Solvent evaporation reduces the temperature of the polymer solution, inducing condensation nucleation and growth at the air–polymer interface. Condensation droplets grow and self-assemble into a hexagonally packed array as the solvent evaporates completely, solidifying the templated polymer film prior to evaporation of the droplets themselves. While the droplet stabilisation and ordering mechanisms in classical BF remain the topic of continuous ongoing investigations, the competing interfacial phenomena of soluto-, thermo- and capillary instabilities, as well as aggregation of a polymer protective layer and solidification are known to suppress droplet coalescence, promoting self-assembly on mono- and multi-layered arrays of highly-packed pores.^13–18^ Total evaporation of the solvent is essential to the creation of the final porous structure, therefore, a specific output pore size and spacing is achieved for given experimental inputs of material properties and environmental conditions.

The facile nature of the classical BF approach lends itself to the passive coupling between the solvent evaporation and polymer curing that foresees both the initiation of condensation and termination of droplet growth with the curing of the pattern.^17–20^ While offering opportunities to create varied self-assembled patterns,^21–23^ this complex interconnection between the dynamic and non-equilibrium condensation/evaporation processes restricts the direct translation of the mechanistic knowledge of drop-wise condensation kinetics^24^ towards predictive design of pore size and spacing in the classical evaporation-driven BF^21,25,26^ approach. Substituting the passive evaporation-driven cooling of the polymer interface with external film cooling, we eliminate the coupling and enable *in situ* interrogation and modulation of the BF patterns through access to discrete intermediate designs within a single dynamic process.

The ability to form a stable BF array is generally governed by the chemical composition and concentration of the polymer/solvent.^21,27–30^ Therefore, full systematic control in the classical BF is fundamentally limited due to the stability constraints of the condensation droplets. If complete solvent evaporation and polymer solidification does not occur fast enough, the droplets will either tend towards coalescence^31–33^ or, given the right conditions, form multi-layer porous structures^14,30,34,35^ due to the sinking of the droplet array. The resultant pore size is influenced by the condensation induction time and the duration/level of subcooling below the dew point, ultimately dictated by the environmental conditions.^36^ Consequently, relatively narrow system-specific working windows are often empirically derived to achieve regular BF patterns at the end of the process, yielding limited systematic control over pore size without substantial modification of material or environmental conditions. Unlike the classical BF approaches where the focus has been on attaining HCP with monodisperse and non-coalescing droplets, in this work we aim to establish direct links with the physics of condensation and evaporation on liquid films to deterministically design monolayer BF patterns of predictable pore size and arrangement.

Recent attempts have been made to further advance the BF pattering approach towards modulating pore shape^22,23,37^ and generating hierarchical structures.^38–40^ The specific pore shape, which is primarily dictated by the interfacial tension at the three-phase contact line, has been altered through the use of non-aqeuous vapors,^22,41^ strict temperature/humidity regulation,^23^ and employing additives^42^ or sacrificial layers.^43^ Secondary patterns have been created *via* consecutive processes such as large-scale re-shaping of the pre-patterned substrates^44^ or BF patterning on physically confined 3D substrates.^39,45–47^ The use of combined techniques with nanoparticles^48–51^ or other self-assembly and phase separation techniques^40,52–54^ have also proven successful for the fabrication of hierarchical BF patterns. The reliance upon hybrid approaches, however, further narrows the range of operational working parameters and choice of materials, impairing the simplicity of the BF method with little to no deterministic control over the final design. Exploiting the reversibility of the condensation and evaporation regimes, we demonstrate that a single process can be implemented to attain monolithic hierarchical BF patterns with bimodal pore size distributions.

**Fig. 1:**
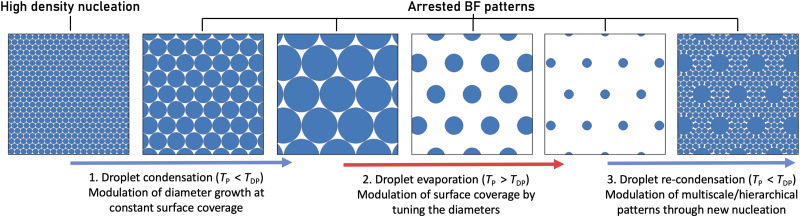
Schematic showing the diversity of patterns hypothesised to occur through subsequent external cooling and heating cycles in the temporally arrested breath figure (BF). The substrate temperature on the Peltier device, *T*_P_, is set with respect to the dew point temperature, *T*_DP_, to alter the patterning regime. (1) When the system is subcooled, the pattern grows in a self-similar regime of constant surface coverage, provided that high density nucleation of water condensation occurs initially. (2) When the system is superheated above the dew point, the droplets shrink radially, decreasing the area fraction covered by the droplet. (3) Re-condensation during a second period of subcooling allows nucleation of new droplet families in the free space between the original condensation sites.

We recently described the adapted temporally arrested BF approach to control the BF pore size systematically using photopolymerisation and external cooling.^55^ Our main goal in this article is to identify control parameters that allow fabrication of temporally arrested BF patterns of predictable pore diameter and spacing through quantitative characterisation of the reversible phase change process. Although reversibility can be practised to some extent in the evaporation-driven breath figure approaches,^23,56^ full reversibility of the condensation process^57^ and access to intermediate patterns are only possible in methods that eliminate solvent evaporation and provide active polymerisation. Here, we explore the possibility of inducing and retracting the reversible BF pattern *via* external temperature control, implementing subsequent regimes of subcooling and superheating with the goal to achieve *in situ* tuning of the size and spatial arrangement of BF patterns, as schematically illustrated in Fig. 1. Further, we investigate the feasibility of restarting the BF process to initiate a second condensation regime, leading to monolithic, multi-scale patterns following a single fabrication route.

## 2 Experimental section

### 2.1 Materials

A commercially available photocurable polymer was chosen due to its fast-curing and oxygen-tolerant cross-linking dynamics. The polymer is a single-component mercapto-ester and acrylate-based system with a viscosity of 2 Pa s (at 25 °C) that was purchased from Norland Optical Adhesives (NOA, Norland Products Inc.) and used as received. Borosilicate glass coverslips (24 mm × 24 mm × 0.15 ± 0.02 mm) were used as thin supporting substrates to fabricate the porous polymer film.

### 2.2 Temporally arrested breath figure patterns

Experiments were performed in ambient laboratory conditions using a simple setup (Fig. S1, ESI†), previously described by Dent *et al.* (2022).^55^ The photocurable polymer was spin coated on a glass coverslip to create thin films of approximately 30 μm thickness. BF patterns were created by placing the coated cover slip directly on the conductive surface of a Linkam PE120 Peltier cooling stage with resolution of ±0.1 °C and cooling/heating ramping rates of 20 °C min^−1^.

The subcooling temperature was set based on the ambient laboratory temperature (*T*_0_) and relative humidity (RH). The subcooling level Δ*T*_C_ = |*T*_DP_ − *T*_P_| was defined as the difference between the set temperature on the Peltier device *T*_P_ and the dew point temperature *T*_DP_ calculated by measuring the ambient conditions before each experiment. Similarly, evaporation of the BF pattern was controlled at a constant superheating level, Δ*T*_H_, with *T*_P_ > *T*_DP_. Supersaturation temperatures between 0 °C to 10 °C prevented freezing (during condensation), while enabling relatively slow kinetics that could be captured by our imaging system and rendered the UV curing time relatively negligible. All reported subcooling and superheating temperatures are reported with respect to the set temperature of the Peltier device. A maximum ±2.5 °C temperature difference between the Peltier surface and glass coverslip was found during the experiments, with negligible temporal-temperature lag during cooling/heating ramps.

To create porous solid surfaces, the liquid BF patterns were arrested and cured at discrete times with a 5 s exposure of 365 nm UV irradiation using a 425 mW cm^−2^ collimated light source (Dymax Redicure550) at 25 mm distance from the substrate.

### 2.3 Pattern characterisation

#### Liquid films

Image analysis of the liquid polymer films was used to elucidate the kinetics of the BF patterns. A long working distance objective (Olympus LMPLFLN 50X) and digital CMOS camera (Basler ace acA2040-90uc) were mounted on an Olympus BX53m optical microscope (OM) providing a nominal spatial resolution of 0.22 μm per pixel. Images were captured at 1 Hz frequency starting from the time at which the coated coverslip was placed on the cooling device, *t*_0_, and characterised using proprietary code and the Image Processing Toolbox in Matlab (Fig. S2, ESI†). The droplet growth progression on the liquid film was analysed through circle identification algorithms to view the average liquid droplet diameter, *D*_L_. Area fraction of droplets, 
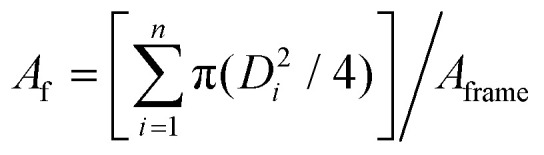
 and polydispersity, 
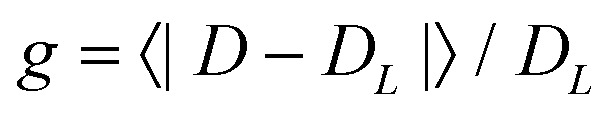
 were calculated based on the individual and average droplet diameters within the field of view. Unless otherwise specified, the image analysis frame and cropped region was set to capture 100's droplets for statistically relevant data, however, the exact size was non-critical due to the low polydispersity (≈10%) across the observation window.

The average interdroplet spacing, *L*_c_, was analysed in different frames captured during the evaporating regime by calculating the average euclidean distance between droplets sharing a vertex in a corresponding Voronoi diagram (Fig. S3, ESI†). Parameters were calculated for every recorded frame and averaged across 10 s intervals to reduce the effects of thresholding errors in individual frames.

#### Cured films

A Hitachi SU8230 scanning electron microscope (SEM) was used at accelerating voltages of up to 5 kV to image the porous solid samples. The diametric measurement on cured films, *D*_S_, refers to the average diameter of the pore opening in SEM analysis. *D*_S_ is smaller than the maximum diameter of submerged droplets (*D*_L_) observable in OM analysis of liquid films (Fig. S4, ESI†). Similar to BF pattern analysis on wet films, area fraction and polydispersity of pores on solid cured samples were calculated using individual and average pore diameters.

## 3 Results and discussion

The pore diameter in the BF pattern can be readily controlled using the temporally arrested BF methodology.^55^ The nucleation and growth of condensation are induced and actively controlled through the application of external cooling, whose elimination will immediately interrupt the growth of water droplets. In the following sections we discuss the quantitative kinetics of condensation droplet growth in a typical temporally arrested breath figure process. We then examine the reversibility of the process, investigating the impact of abruptly changing the substrate temperature to values above the dew point and quantifying the kinetics of droplet evaporation. Through *in situ* control of condensation and evaporation, we finally propose and test a protocol to attain hierarchical BF patterns.

### 3.1 Condensation: modulation of droplet size at constant surface coverage

Under quiescent conditions of constant temperature and no significant airflow, the growth rates of droplets forming the BF pattern follow the well-described dew condensation physics^24^: for isolated droplets, diffusion-limited growth increases the droplet volume monotonically, leading to a diametric growth relationship of *D*_L_ ∝ *t*^1/3^.^58^ However, when droplets pack closely, their growth is accelerated due to the coalescence between neighbours, yielding a self-similar growth regime in which droplet diameters follow *D*_L_ ∝ *t*.^36^

**Fig. 2:**
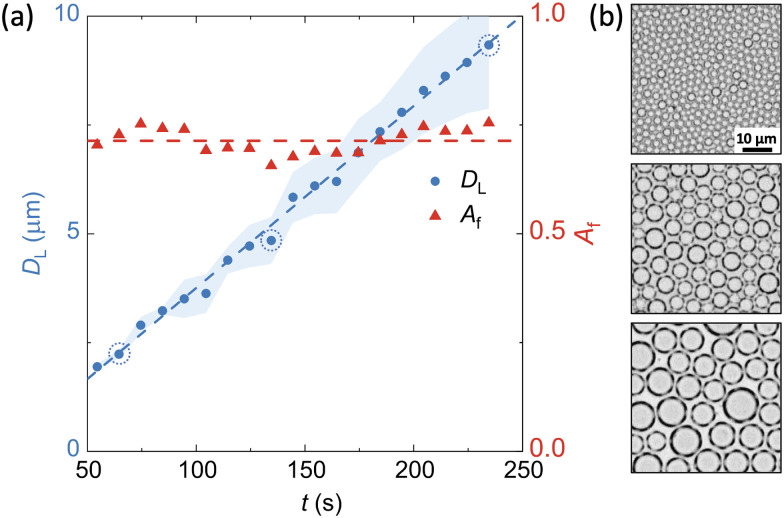
BF patterns captured on NOA63 with high initial nucleation density at *T*_0_ = 19 °C, RH = 70% and Δ*T*_C_ = 10 °C. (a) Real-time temporal evolution of the condensation droplet diameters following a *D* ∝ *t* trend line (*R*^2^ coefficient of 0.99) and reported standard deviation. *A*_f_ remains near-constant throughout the process. (b) OM images of early, intermediate and late-stage droplet growth (circled on the graph) demonstrate the self-similarity of the BF patterns.

Under optimised material and environmental conditions, stabilised condensation droplets in classical BF grow mainly by diffusion, forming monodisperse HCP with negligible coalescence between droplets.^26^ In contrast, the condensation droplets in our BF patterns appear at high initial nucleation density and grow due to diffusion and significant coalescent events. Fig. 2a shows the kinetics of droplets’ growth on the NOA63 photocurable polymer film which was previously found to promote high-density nucleation.^55^ Deterministic control of the droplet size in the BF patterns is achieved by maintaining a constant subcooling level at temperatures below the dew point. By permitting droplet coalescence, high packing can be maintained within a single layer BF pattern with no upper limit for the droplet size.^14^ Although disturbing the hexagonal packing order and inducing polydispersity, the coalescent-driven growth allows systematic control of pore diameter in a repeatable and predictable manner.

The monotonic growth rate shows conformation to the linear trendline of *D* ∝ *t* with visible highly packed and self-similar condensation growth (Fig. 2a). In this coalescent-dominated regime, the area fraction of condensation droplets remains constant at *A*_f_ = 0.71 ± 0.05, demonstrating near optimal packing when accounting for the slight disorder due to the droplet polydispersity and the thin viscous polymer film stabilising the droplets, as observed in Fig. 2b.^59^ While variation in the droplet diameters occurs due to coalescence between neighbouring droplets, the polydispersity throughout the analysis in this regime remains low and near constant at *g* = 0.10 ± 0.05. This range is similar to that found in original studies on non-stabilised BF droplet growth on liquid films,^58^ whilst being within the ranges reported in non-HCP^21,25,60,61^ BF patterns and late-time condensation regimes.^62^ The self-similar dynamic BF pattern on the liquid polymer film (Fig. 2b) can be arrested *via* UV irradiation at any moment of time to achieve a patterned surface of known pore diameter and area coverage. Transitioning from droplets in liquid films to pores in the cured polymer, there is no significant change in the shape across the experimental tests or after UV exposure due to the largely dominant interfacial forces at these length scales.

### 3.2 Evaporation: modulation of surface coverage at constant interdroplet spacing

**Fig. 3:**
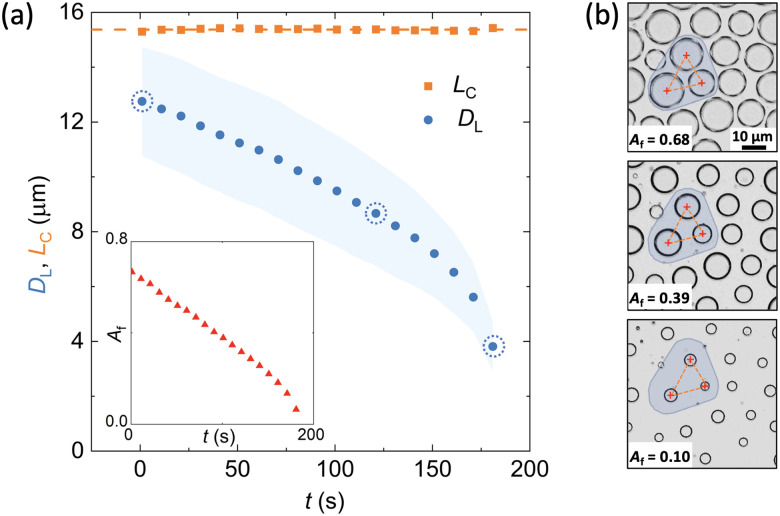
Evolution of BF patterns during the evaporation regime with *t*_0_ taken as the time at which the correct superheating temperature was reached. Data was collected at *T*_0_ = 22 °C, RH = 55% and Δ*T*_H_ = 5 °C. (a) In the evaporation regime, the droplet diameter decreases while the interdroplet spacing remains constant. Shaded area refers to the standard deviation. (b) OM images from initial, intermediate and late stage analysed times (circled on the graph) show the constant interdroplet spacing created from the previous condensation regime.

At a given relative humidity, the difference between the substrate temperature and the dew point sets the supersaturation level and controls the rate of phase change. For set temperatures below the dew point (*T*_P_ < *T*_DP_), the subcooling defines the linear growth of BF droplets in time during the BF formation (Fig. 2). Conversely, for set temperatures above the dew point (*T*_P_ > *T*_DP_) on samples with a well-developed BF assembly, condensation is stopped, followed by the evaporation of the droplets (Fig. 3).

Using our real-time optical microscopy analysis, we characterise the evaporation kinetics of droplets within BF patterns formed on NOA63 at constant superheating levels. While in the highly packed condensation regime, droplets are being pushed by the neighbours and rearrange due to coalescence, they remain fairly stationary in the evaporation regime indicating negligible capillary interactions or droplet diffusion in the length and time scales studied here. During evaporation, the radially shrinking droplets maintain a constant interdroplet spacing, *L*_c_ ≈ 1.2 × *D*_L_0__, with *D*_L_0__ the maximum droplet diameter attained in the highly packed condensation regime (Fig. 3a). Unlike in the condensation regime where the area fraction covered by the BF pattern remains unchanged, evaporation leads to continuous reduction in the droplet diameters whilst the distance between them is maintained; the area fraction can thus be significantly reduced in a controlled manner during the evaporation regime by understanding the evaporation kinetics. In the example shown in Fig. 3, the area fraction covered by the BF pattern decreases by more than 85% from the initial value of *A*_f_ = 0.68.

In cured BF patterns, while pore size modulation at constant high surface packing was achieved *via* temporally arrested BF, the evaporation step allows diameter modulation of the equally spaced pores, reducing the area fraction.

**Fig. 4:**
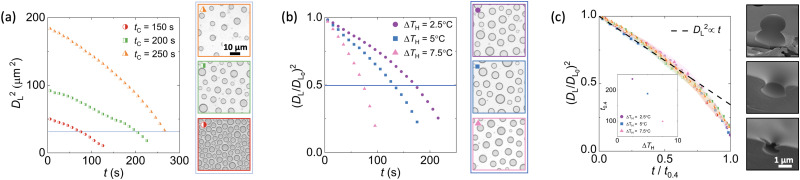
Kinetics of BF evaporation analysed for individual droplets and averaged over 10 s intervals, see Fig. S5 in the ESI† for more details. (a) The square of droplet diameter monotonically decreases with time. The initial average diameter in the fully packed BF is controlled by varying the duration of the condensation regime *t*_c_, before starting the superheating cycle. Data corresponds to Δ*T*_H_ = 5 °C, *T*_0_ = 19 °C and RH = 66%. Snapshot images show patterns with similar average square diameter *D*_L_^2^ = 30 μm^2^. (b) Higher levels of superheating speeds up the BF pattern evaporation. Snapshots show patterns with similar square normalised diameters (*D*_L_/*D*_L_0__)^2^ = 0.7 and area fractions, obtained at different superheating levels. Data was captured at *T*_0_ = 22 °C, RH = 55% and the respective Δ*T*_H_ detailed on the graph. (c) Normalising pattern diameters and time allows the collapsing of all data presented in (a) and (b). SEM images correspond to the morphology of a typical BF pore (top), and sinking pores observed at late-stage of the evaporation cycle (middle and bottom).

Droplet diameter continuously decreases during the evaporation, suggesting that evaporation of BF patterns on a liquid substrate occurs in a constant contact angle regime, where the droplets maintain a spherical cap with diminishing drop height and contact area. Previous theoretical and experimental investigations confirmed that the mass, *M*, of a sessile droplet evaporating on hydrophobic substrates in isothermal conditions decreases according to *M*^2/3^ ∝ *t*. The relationship is derived in the regime where the contact line recedes without significant change in the contact angle.^63,64^ Relating the droplet mass to its diameter *D* predicts *D*^2^ to decrease linearly with time during the evaporation if the droplet shapes remain self-similar. To further investigate the viability of the combined condensation and evaporation cycles for pattern modulation and the validity of the constant contact angle regime for droplet evaporation, analysis of the time-dependent evolution of evaporating BF patterning droplets for a range of operating and environmental conditions is performed. In order to eliminate any smearing effect caused by size polydispersity (Fig. S5, ESI†) and gain a better understanding of the evaporation mechanism, individual droplets are tracked and analysed in Fig. 4, see more details in the ESI.† All data is presented in square diameter to allow comparison with the available prediction models.

For a given Δ*T*_C_ and RH, *L*_c_ is only a function of the time from the start of the BF formation in the highly packed condensation process, *t*_C_. Therefore, combining the condensation and the evaporation steps provides a powerful handle for dynamic spatial modulation of BF patterns where both droplet diameter and area fraction can be controlled. Initiating the evaporation at different times from the start of the condensation nucleation allows the generation of BF patterns with different diameters and interdroplet spacing for given subcooling and superheating conditions. Fig. 4a shows diverse patterns with similar average droplet diameters but varying interdroplet spacing obtained by tuning *t*_C_. The snapshot images correspond to *D*_L_^2^ = 30 μm^2^, showing that BF patterns can be arrested with different arrangements and area coverage at constant pore diameters. Additional control over the evaporating BF pattern is obtained by adjusting the level of superheating set by the value of Δ*T*_H_, *i.e.* higher superheating levels yield faster evaporation and smaller final diameters with larger interdroplet spacing at a given time from the start of the evaporation cycle. Constant average pattern diameter and interdroplet spacing can thus be obtained by arresting varying superheated evaporation cycles at different times, creating effectively identical patterns using different input parameters, see example snapshots of patterns in Fig. 4b.

While the trends in droplet diameter reduction appear to be similar in Fig. 4b, higher levels of superheating yield faster shrinking of the droplet. Normalising the *x*-axis by an arbitrary time, *t*_0.4_, at which the average droplet diameter reaches 40% of its initial value (*D*_L_/*D*_L_0__ = 0.4), accounts for the impact of superheating and in turn collapses all data captured at varied operational and environmental conditions onto a single curve (Fig. 4c). A linear trend, (*D*_L_/*D*_L_0__)^2^ ∝ *t*/*t*_0.4_, provides a fair prediction of the collapsed data until (*D*_L_/*D*_L_0__)^2^ = 0.5 is reached. At later times we observe a significant increase in the droplet shrinkage rate with deviation from the predicted linear trend (Fig. 4c). At these times, some of the condensation droplets in the field of view appear out of focus, suggesting an increasing frequency of droplets moving downward relative to the top interface of the polymer film.^65^ This vertical translation is clearly observed in the SEM images of the cured films’ cross sections presented in Fig. 4c, where droplets sink below the interface and eventually become fully encapsulated within the polymer, see further details in Fig. S6 in the ESI.† As we employ the BF approach for surface patterning, later evaporation times where initial water droplets are no longer all on the surface are not considered for further analysis. Compared to the condensation regime, the polydispersity in droplet diameters gradually increases to *g* = 0.20 ± 0.05, up to a maximum value of *g* ≈ 0.3 at the final stages of evaporation where the downward translation of droplets is observed. This increase arises from the initial polydispersity in the highly packed droplet population, augmented by the different evaporation rates and vertical translation of droplets within the field, especially at smaller diameters (Fig. 4c).

### 3.3 Re-condensation: hierarchical patterns in combined condensation-evaporation regimes

Dynamic droplet patterns are transformed into static porous designs only after UV exposure of the photopolymer in the temporally arrested BF approach. Therefore, the reversible condensation and evaporation regimes can be arranged in repeated cycles to attain diverse BF architectures prior to polymer curing.^57^ The evaporation regime creates free surface area on the polymer film interface by reducing the area fraction of BF pattern. These plain areas can be subsequently re-patterned by generating a new population of droplets as we cool the polymer film below the dew point again, restarting the condensation phase. In this post-evaporation regime of condensation, original droplets grow simultaneously with the newly nucleated droplets to create a bimodal distribution of the templating droplets, thus allowing the development of hierarchical BF designs.

**Fig. 5:**
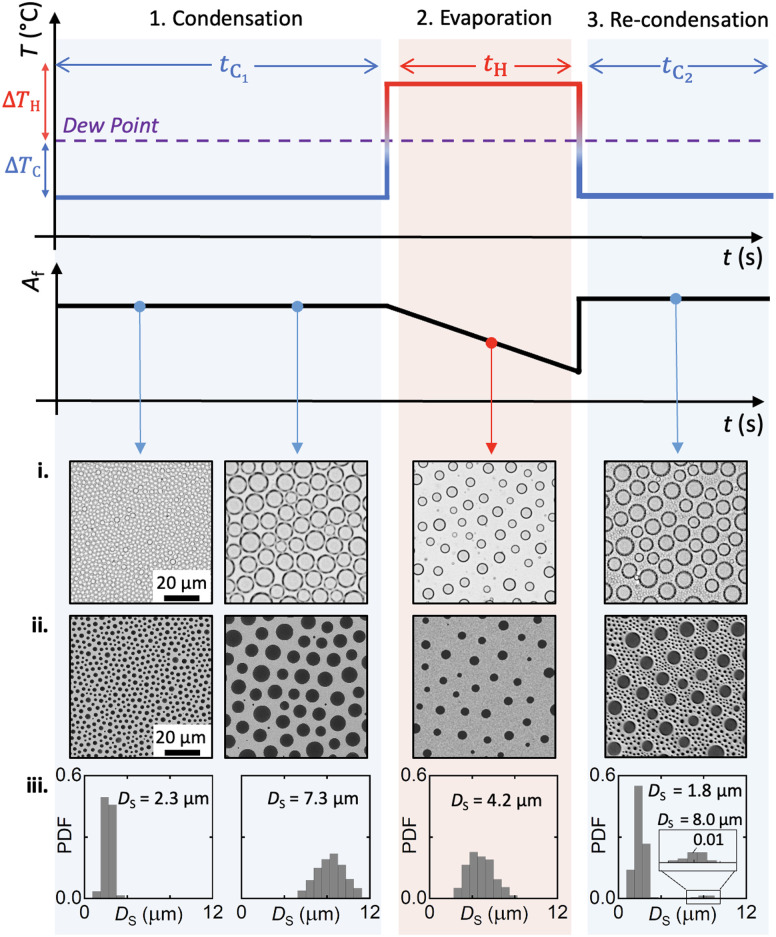
Schematic illustration of the complete experimental process followed for hierarchical BF patterning, demonstrating the modulation of temperature around the system dew point and examples of respective patterns obtained. Data was collected at *T*_0_ = 22 °C, RH = 36%, Δ*T*_C_ = 10 °C (*t*_C_1__ = 250 s, *t*_C_2__ = 140 s) and Δ*T*_H_ = 5 °C (*t*_H_2__ = 250 s). (i) Real-time OM images capturing the maximum diameters of condensation droplets below the interface of the polymer film with air. (ii) SEM images of samples cured at the discrete times showing the pores’ openings on the surface of the polymer film. (iii) Probability Density Function of the pore diameters, derived from the SEM images.

Fig. 5 schematically illustrates the experimental procedure and real-time microscopy images of the BF patterns produced through a simple combined cycle of condensation, evaporation and re-condensation: (1) an initial subcooling period of condensation is completed to establish highly packed BF patterns. This regime dictates the specific interdroplet spacing for the large droplet domains as previously elucidated. (2) The system is then superheated to reduce the droplet packing as the droplet diameters decrease during the evaporation regime. (3) Restarting the subcooling regime for a second time, we initiate the re-condensation regime manifested by the appearance of new condensation nucleation sites on the free surface of the polymer, as well as the growth of the original droplets.

SEM images of the cured films and the corresponding histograms of pore diameters are presented in Fig. 5(ii and iii), respectively. The visible diameters on the liquid (i) and solid (ii) films differ as they are associated with the full droplet diameter visible during liquid analysis and the pore opening diameter visible with SEM (Fig. S4, ESI†). The pattern histograms (iii) show the polydispersity in pore diameters and their distribution at different times. The active regulation of the substrate temperature allows us to control the interdroplet spacing in the primary regime and ultimately modulate the area fraction to create cured porous surfaces of desired characteristics. Through consecutive regimes of cooling and heating, we harness the predictable kinetics and reversibility of the temporally arrested BF patterning approach to achieve multiscale structures. Optical microscopy video of a continuous condensation, evaporation and re-condensation can be viewed in the ESI.†

## 4 Conclusions

Biological patterned surfaces exhibit a wide range of patterns and morphologies created through energy-efficient and self-assembling means. While effectively functional, these inspirational designs have a natural tolerance to imperfections and defects, affording engineered counterparts not to require the high-fidelity and costly fabrication routes of typical cleanroom and mask-driven approaches. Here, we demonstrated a simple biomimetic fabrication route for scalable and low-cost manufacturing of diverse programmable patterns using a single material and process.

The current temporally arrested BF method provides dynamic, *in situ* control over the size and spatial distribution of interfacial pores in polymer films. We explored the inherent reversibility of the phase change mechanism governing the process prior to curing of the polymer. Condensation and evaporation kinetics on the liquid films were analysed in controlled regimes of subcooling and superheating, respectively, modulating the average droplet size at constant surface coverage before modulation of the surface coverage. Ultimately, hierarchical and multiscale patterns were formed through combined condensation–evaporation cycles.

**Fig. 6:**
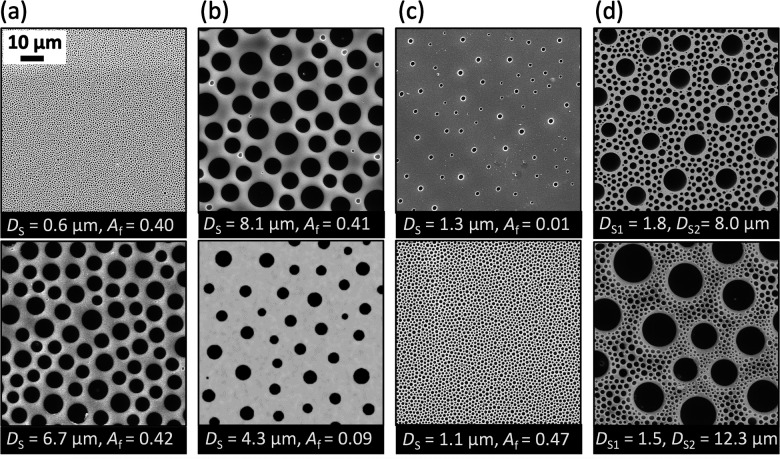
SEM images of the cured patterns attainable from the temporally arrested BF process, showing the diversity of the programmed design. (a) Constant area fraction at varying diameters. (b) Constant interdroplet spacing at varying diameters. (c) Constant diameters at varying interdroplet spacing. (d) Varied hierarchical designs. Operational parameters are included in Table S1 in the ESI.†

An example catalogue of the cured designs is presented in Fig. 6; diverse patterns are created with constant packing but an order of magnitude differing diameters (Fig. 6a), constant interdroplet spacing at varying diameters (Fig. 6b), constant diameters at differing area fractions (Fig. 6c), and finally bimodal distributions of pore diameters (Fig. 6d). The full BF reversibility for surface patterning practised here is a unique feature of the temporally arrested approach thanks to the external temperature control, rapid UV curing and the elimination of solvent evaporation. Further control over the BF pattern and pore morphologies can be achieved through tailoring properties of the photocurable polymer^66^ and finely tuning the environmental parameters^23^ to facilitate viable commercial and industrial exploitation of the method.

## Conflicts of interest

There are no conflicts to declare.

## Supplementary Material

SM-019-D2SM01650H-s001

SM-019-D2SM01650H-s002
