# Supplementary material for: Exploiting breath figure reversibility for in situ pattern modulation and hierarchical design
Source: Soft Matter. 2023 Mar 14;19(15):2737–44. doi: 10.1039/d2sm01650h (PMC10091834; doi:10.1039/d2sm01650h)
Supplement: SM-019-D2SM01650H-s001 [file SM-019-D2SM01650H-s001.pdf]

Electronic Supplementary Material (ESI) for Soft Matter.
This journal is © The Royal Society of Chemistry 2023

Supporting Information
Exploiting breath figure reversibility for *in-situ* pattern modulation and hierarchical design

Francis J. Dent[1], David Harbottle[2], Nicholas J. Warren[2], and Sepideh Khodaparast[1,*]

[1]School of Mechanical Engineering, University of Leeds, LS2 9JT Leeds, UK.
[2]School of Chemical and Process Engineering, University of Leeds, LS2 9JT Leeds, UK.
[*]email: s.khodaparast@leeds.ac.uk

List of Supporting Materials

Figure S1. Experimental setup.
Figure S2. Droplet identification protocol.
Figure S3. Interdroplet spacing analysis.
Figure S4. Observable features in diametric analysis.
Figure S5. Evaporation analysis: full film average diameter vs individual droplet.
Figure S6. Downward translation of droplets during evaporation.
Figure S7. Experimental operating parameters.
Video S1. Optical microscopy video of a full condensation, evaporation and re-condensation cycle.

S1 Experimental setup

All experiments were performed in open laboratory conditions without further controlling the humidity or the temperature. A thin film of NOA63 was spin coated on a glass cover slip. The thickness of the polymer film and the cover slip were approximately 30 μm and 150 μm, respectively. The coated coverslip was directly located on the smooth conductive surface of the Peltier stage, see Fig. S1. The dew point was calculated according to the relative humidity RH and temperature T_0 of the environment before every test. The sub-cooing and the super-heating levels were then set by regulating the temperature of the Peltier device. Up to ±2.5°C temperature difference was measured between the surface of the Peltier stage and the top surface of the glass cover slip.

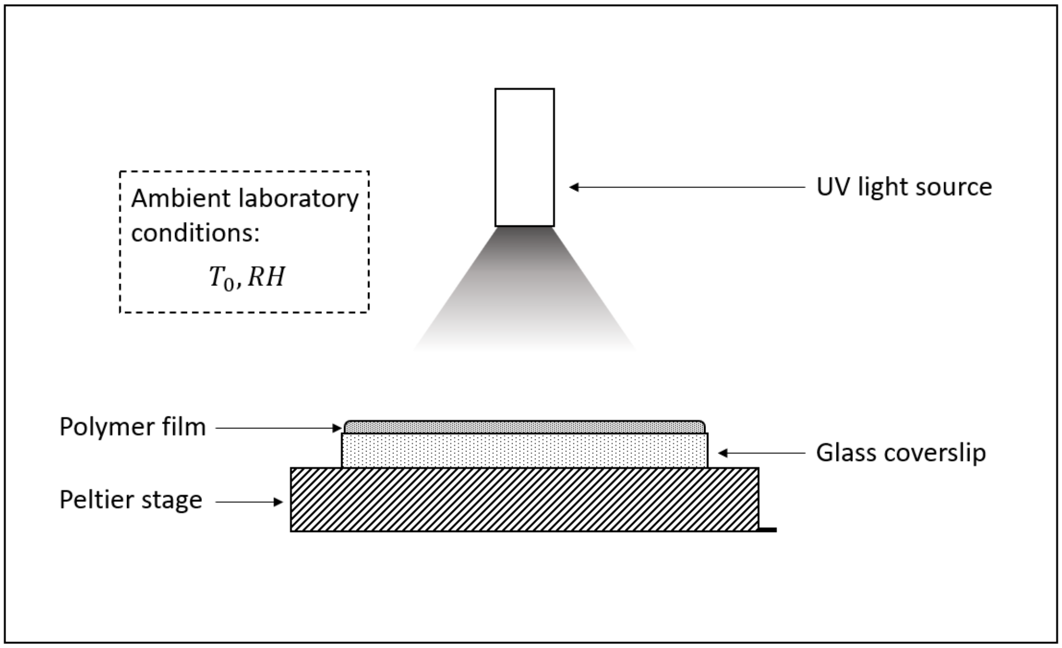

Figure S1: Schematic of the temporally arrested breath figure experimental setup.

S2 Droplet identification protocol

Droplet identification was used as the predominant mode of analysis for parameters in this paper. Analysis was completed on both raw optical microscopy images (a) of the liquid samples and SEM images of the cured samples. The circular Hough transform function on Matlab was used to detect droplet interface (b) as described in Fig. S2. Thresholded image (c) was used to calculate the area fraction while image (d) with the exclusion of partially occluded droplets was used to calculate the average droplet diameter and polydispersity parameters.

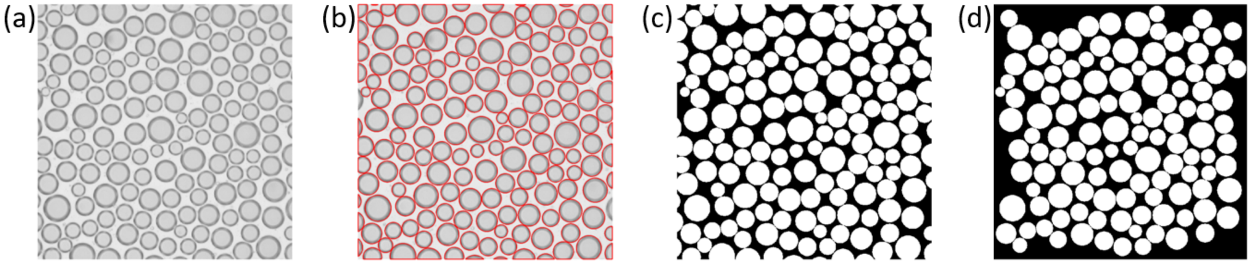

Figure S2: (a) Raw images were cropped to negate uneven lighting and lens distortion. (b) Circles were identified using the circular Hough transform command on Matlab. (c) The image was binarised. (d) The edge droplets were negated from the final image.

S3 Interdroplet spacing analysis

The spatial distribution of droplets was analysed by calculating the average interdroplet spacing using Matlab. Voronoi diagrams (blue outline) were plotted for respective images after the central points (red crosses) were found via the previous thresholding method (Fig. S2). The average Euclidean distance was calculated by computing the distance between a droplet centre and the neighbouring droplet and averaged across all the droplets in the frame. Neighbouring droplets were identified as sharing an edge in the corresponding Voronoi plot (Fig. S3).

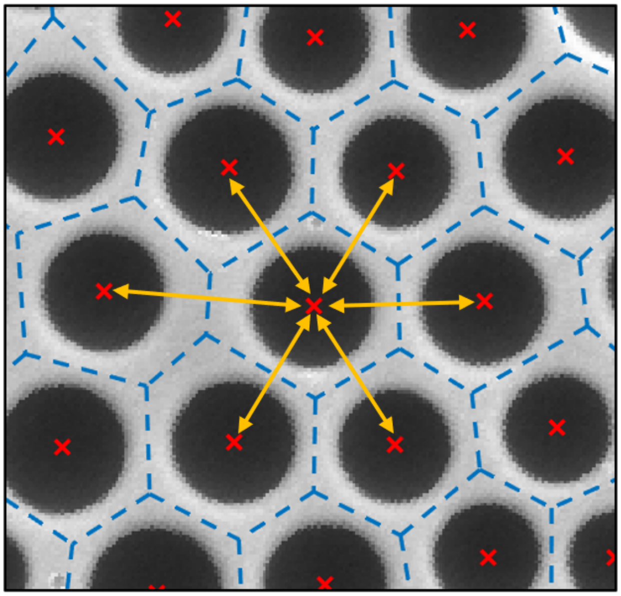

Figure S3: Interdroplet spacing, L_C, for the droplet pore in the centre is defined as the average Euclidean distance with the neighbouring droplets.

S4 Observable features in diametric analysis

Analysis of the liquid and solid BF patterns reveals different diameters. Due to the relative refractive indices upon analysis of liquid BF using optical microscopy from above, the widest diameter (D_{L}) can be observed. In SEM analysis of solid patterned films, the pore top diameter at the surface (D_{S}) is observed.

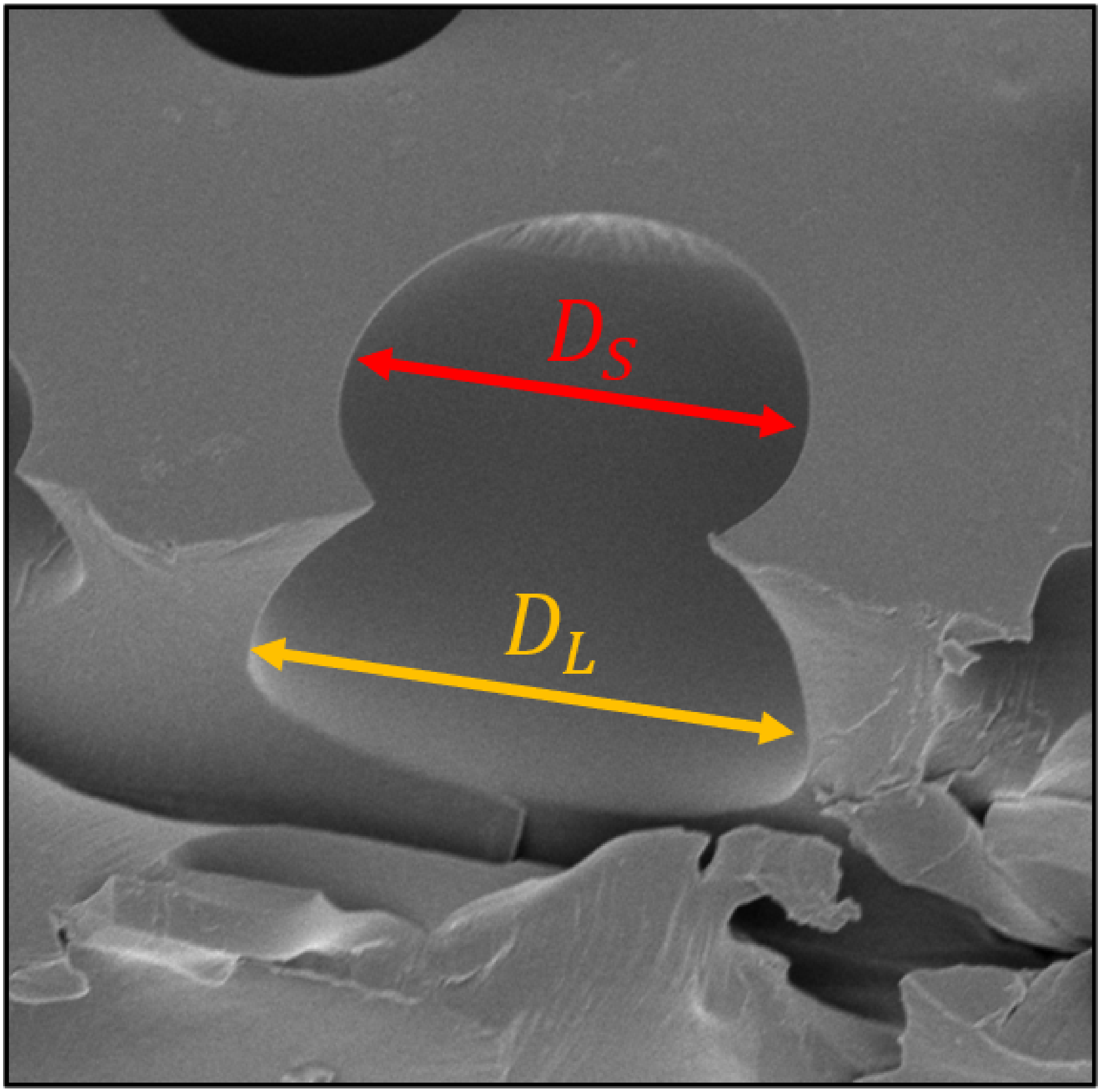

Figure S4: SEM cross-section of a pore in a cured sample showing both the pore opening on the surface and largest diameter underneath the surface.

S5 Evaporation analysis: full film average diameter vs individual droplet

As the polydispersity in droplet diameter during the evaporation smears the trends especially at later times, a single randomly selected droplet in Fig. 4 is tracked to investigate the evaporation kinetics of condensation droplets in the system. Fig. S5 shows the smearing effect observed when comparing the reduction in droplet diameter for a single droplet compared to full-field analysis. To ensure that the randomly selected droplet statistically represents the kinetics across the field of view, four other droplets of similar diameters (with maximum diameter variation of ± 5%) were selected from around the field of view at the start of the analysis. Identical droplets were then followed during the evaporation regime. All data presented in Fig. 4a, b and c demonstrate average values calculated for these five droplets.

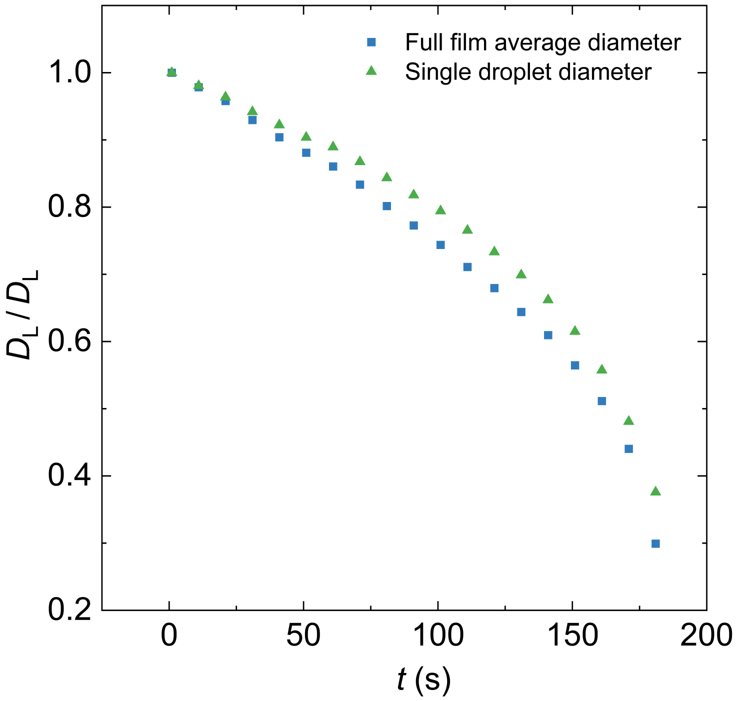

Figure S5: Data showing the standardised droplet diameter in the evaporation regime. The full film average diameter is plotted vs the average of five identical droplets.

S6 Downward translation of droplets during evaporation

SEM analysis of a sample cured at a later time during the evaporation cycle. By reducing the accelerating voltage on the SEM from 5 kV (a) to 0.1 kV (b), a number of droplets that are encapsulated right underneath the surface disappear from the view. These droplets were fully observable in the OM analysis, and their diametric analysis is included in Fig. 4c.

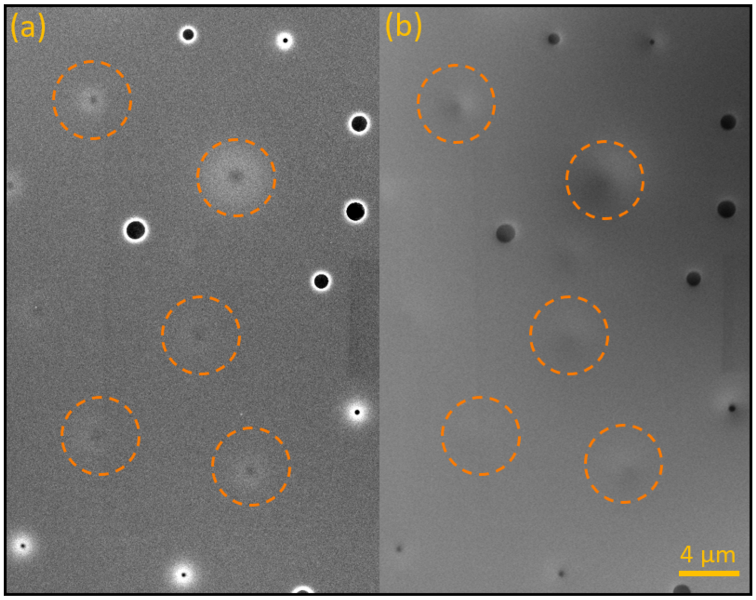

Figure S6: SEM images of a late-stage evaporation sample showing highly dispersed droplets. Sample imaged at (a) 5 kV and (b) 0.1 kV show droplets encapsulated by a thin polymer film right below the interface.

S7 Experimental operating parameters

Fig. 6 in the main article presents SEM examples of 8 diverse breath figure samples obtained following different cooling and heating pathways. A summary of the environmental relative humidity RH and temperature T_0, the subcooling and superheating temperatures and their corresponding duration is presented in the table below. t_{C_1} and t_{C_2} refer to the duration of the first and the second condensation regimes at ΔT_C, while t_H refers to the duration of the evaporation regime at ΔT_H. Note that the same sample morphology can be created using any number of parameters as the pattern evolution rate is inferred from the supersaturation levels. The changes in T_0 and RH reflect the changes in the ambient laboratory conditions.

Table S1: Table of parameters for Fig. 6 samples.

Sample	T_0 (°C)	RH (%)	ΔT_C (°C)	t_{C_1} (s)	t_{C_2} (s)	ΔT_C (°C)	t_{H_1} (s)
A1	22	63	10	20	-	-	-
A2	22	63	10	180	-	-	-
B1	23	57	10	230	-	-	-
B2	19	70	10	240	-	5	500
C1	23	54	10	300	-	5	360
C2	22	63	10	30	-	-	-
D1	19	70	10	240	225	5	280
D2	20	68	10	360	240	5	300
